# Supplementary material for: Pyrene-Based Fluorescent Porous Organic Polymers for Recognition and Detection of Pesticides
Source: Molecules. 2021 Dec 26;27(1):126. doi: 10.3390/molecules27010126 (PMC8746865; doi:10.3390/molecules27010126)
Supplement: Supplementary file 1 [file molecules-27-00126-s001.zip › molecules-1466133-supplementary.pdf]

Supplementary information

Pyrene-Based Fluorescent Porous Organic Polymers for Recognition and Detection of Pesticides

Zhuojun Yan,[1] Jinni Liu,[1] Congke Miao,[2] Pinjie Su,[2] Guiyue Zheng,[1] Bo Cui,[1] Tongfei Geng,[1] Jiating Fan,[1] Naishun Bu,[2,*] Ye Yuan,[3,*] and Lixin Xia[1,4,*]

[1] College of Chemistry, Liaoning University, Shenyang 110036, China; zjyan@lnu.edu.cn (Z.J.Y.); ljn1556939707@163.com (J.N.L.); zhengguiyue1996@163.com (G.Y.Z.); cuibo2019@163.com (B.C.); mgpanda95108@163.com (T.F.G.); fjt123465789@163.com (J.T.F.); Yzy20010313@163.com (Z.Y.Y.)

[2] School of Environmental Science, Liaoning University, Shenyang 110036, China; miaocongke@163.com (C.K.M.); spj2580@163.com (P.J.S.)

[3] Key Laboratory of Polyoxometalate and Reticular Material Chemistry of Ministry of Education, Faculty of Chemistry, Northeast Normal University, Changchun 130024, China.

[4] Liaoning Key Laboratory of Chemical Additive Synthesis and Separation, Yingkou Institute of Technology, Yingkou 115014, China.

* Correspondence: bunaishun@lnu.edu.cn (N.S.B.); Yuany101@nenu.edu.cn (Y.Y.); lixinxia@lnu.edu.cn (L.X.X.)

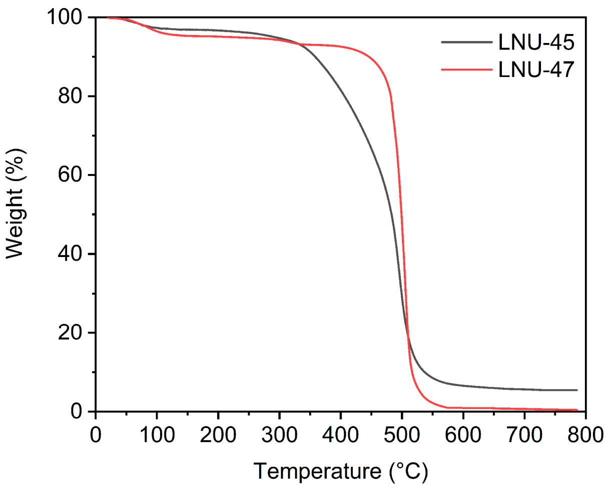

Figure S1. TGA plots of LNU-45 and LNU-47 at the air condition.

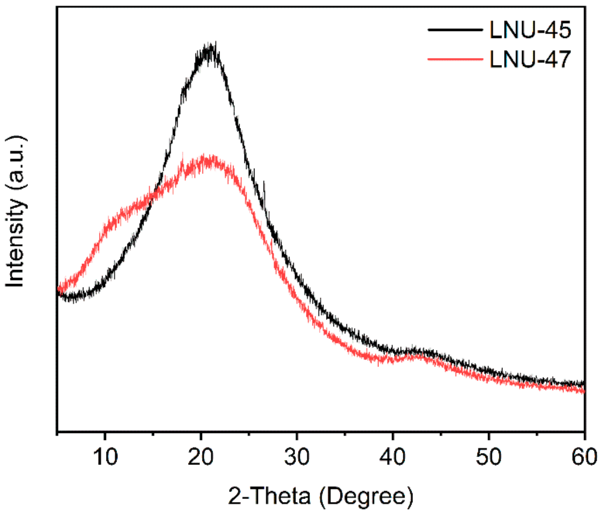

Figure S2. Powder X-ray diffraction patterns of LNU-45 and LNU-47.

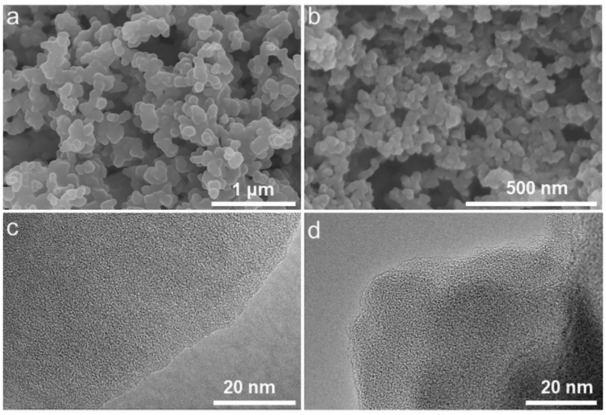

Figure S3. SEM images for LNU-45 (**a**) and LNU-47 (**b**). TEM images for LNU-45 (**c**) and LNU-47 (**d**).

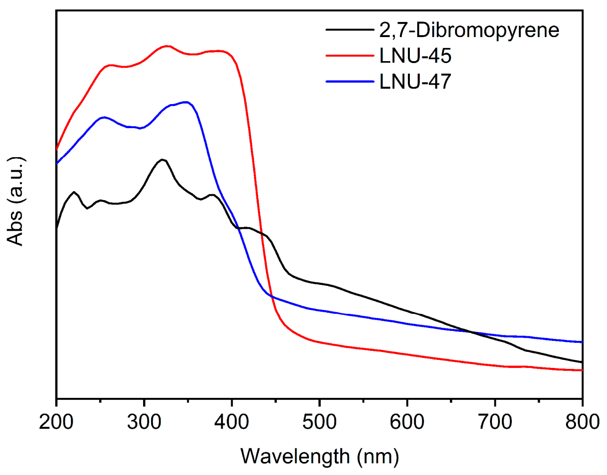

Figure S4. Solid UV-visible absorption spectra of 2,7-dibromopyrene, LNU-45 and LNU-47, respectively.

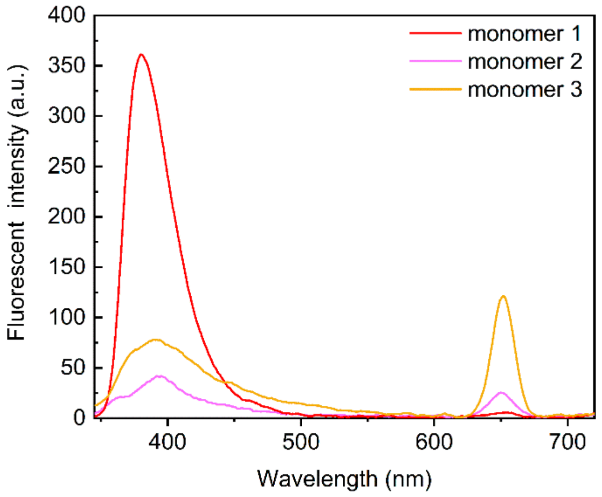

Figure S5. Fluorescence spectra of tris(4-boronic acid pinacol ester phenyl)amine (monomer 1), 2,7-dibromopyrene (monomer 2) and benzene-1,3,5-triyltriboronic acid pinacol ester (monomer 3), respectively.

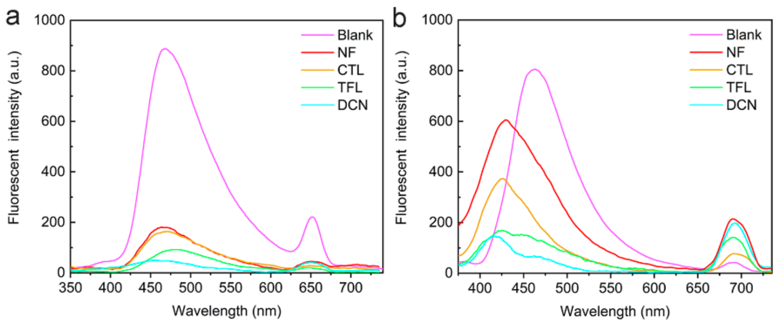

Figure S6. (**a**) Fluorescence spectra for the suspensions of LNU-45 (0.25 mg mL^{-1}) with different pesticides. (**b**) Fluorescence spectra for the suspensions of LNU-47 (0.25 mg mL^{-1}) with different pesticides.

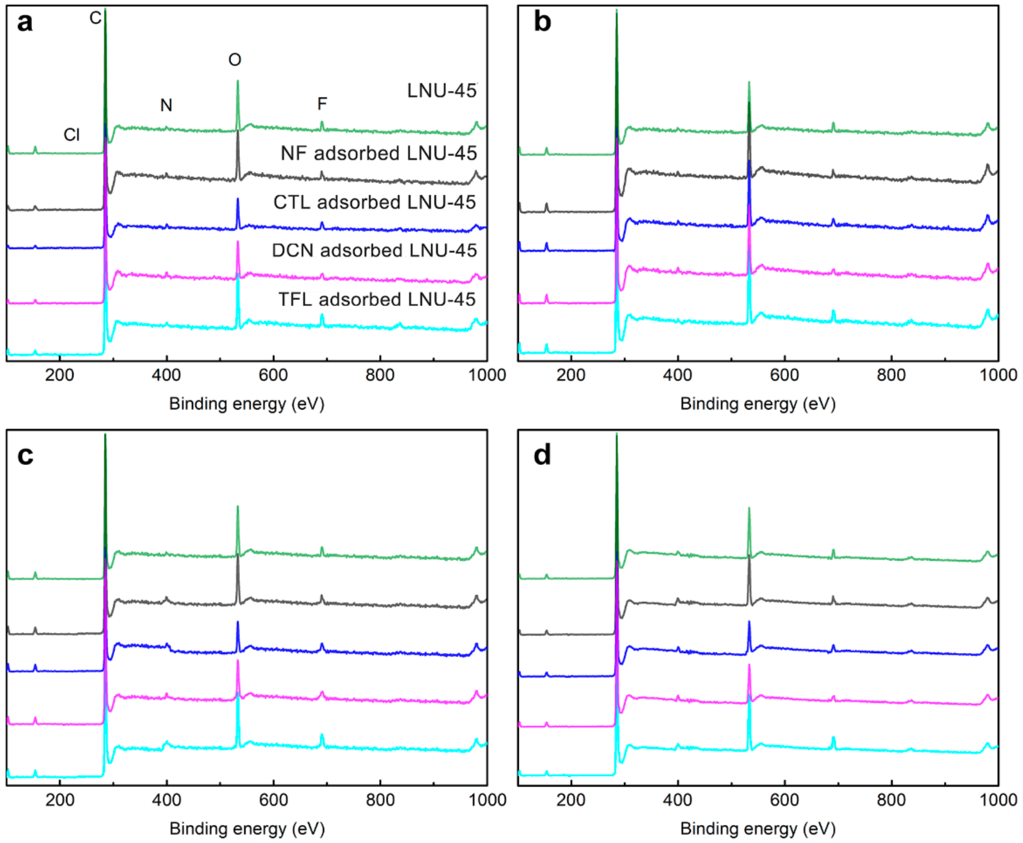

Figure S7. Raw XPS spectra for POP sample and pesticide adsorbed POP sample (**a**). XPS spectra for POP sample and pesticide adsorbed POP sample with a self-consistent (LNU-45 derived) integral baseline (**b**), a self-consistent (LNU-45 based) integral baseline (**c**) and a self-consistent (average) integral baseline (**d**).

X-ray photoelectron spectroscopy (XPS) and solid-state ^{13}C NMR spectroscopy are advanced technologies in the microscopic analysis of electronic materials and components, which provides information on elemental composition and content [1,2]. Herein, they are utilized to determine the relative content of some difficult-to-measure substances, in this article fluorine and chlorine. However, there are so many cases where the background baseline will seriously affect the analysis results [3]. Scientists have conducted in-depth research on it, and want to propose accurate methods for assessing peak area and relative intensity, such as: Shirley, Linear, Touggard, and other background baseline subtraction methods [4-7]. Based on the above considerations, we wanted to explore a normal strategy to build a self-consistent integral baseline, which is helpful for the calculation of the relative content of each element (C, N, F, Cl) corresponding to the amount of pesticide adsorbed in POPs architecture.

For Figure S7b, the self-consistent integral baseline is obtained by using LNU-45 as the object. We performed a noise reduction process on the baseline of the pesticide adsorbed POP materials (the noise reduction amplitude is 200%). The reduced noise of the pesticide adsorbed POP material is then added to that of LNU-45 to obtain the

LNU-45 derived the self-consistent (LNU-45 derived) integral baseline. For Figure S7c, the self-consistent integral baseline is obtained by using LNU-45 as the standard, we replace the baselines of other LNU samples by that of LNU-45 as the self-consistent (LNU-45 based) integral baseline. For Figure S7d, the self-consistent integral baseline is obtained by adding the baseline of 5 sets of data, and then divide by 5 to get the average baseline as the self-consistent (average) integral baseline.

As shown in Figure S7, the C/N ratio in Table S4 is similar with that the CHN analysis (Table S5), proving that the element content calculated by using the self-consistent (average) integral baseline is more accurate. Accordingly, the Cl contents in pesticide adsorbed POP samples are 28.6 mg g^{-1} (NF adsorbed LNU-45), 37.1 mg g^{-1} (CTL adsorbed LNU-45), and 46.6 mg g^{-1} (DCN adsorbed LNU-45); and the F content in TFL adsorbed LNU-45 is 45.2 mg g^{-1}. Correspondingly, the adsorption uptakes are calculated to be 116.0 mg g^{-1} (NF adsorbed LNU-45), 70.0 mg g^{-1} (CTL adsorbed LNU-45), 139.8 mg g^{-1} (DCN adsorbed LNU-45), and 231.9 mg g^{-1} (TFL adsorbed LNU-45).

Table S1. Element analysis based on the integral area of XPS spectra (Figure S7a).

	Element	Cl_{2p}	C_{1s}	N_{1s}	F_{1s}	C/N
	Binding energy (eV)	189 – 202	280 – 295	395 – 410	680 – 695	ratio
LNU-45	Integral area	1916	413817	24267	40231	
	Relative content	0.40%	86.17%	5.05%	8.38%	17.06
NF adsorbed	Integral area	18573	507635	37214	34512	
LNU-45	Relative content	3.11%	84.90%	6.22%	5.77%	13.65
CTL adsorbed	Integral area	14046	347552	35891	41475	
LNU-45	Relative content	3.20%	79.18%	8.17%	9.45%	9.69
DCN adsorbed	Integral area	17542	476884	35748	41719	
LNU-45	Relative content	3.07%	83.39%	6.25%	7.29%	13.34
TFL adsorbed	Integral area	1475	495438	28658	78425	
LNU-45	Relative content	0.24%	82.03%	4.75%	12.98%	17.27

Table S2. Element analysis based on the integral area of XPS spectra (Figure S7b).

	Element	Cl_{2p}	C_{1s}	N_{1s}	F_{1s}	C/N
	Binding energy (eV)	189 – 202	280 – 295	395 – 410	680 – 695	ratio
LNU-45	Integral area	1916	413817	24267	40231	
	Relative content	0.40%	86.17%	5.05%	8.38%	17.06
NF adsorbed	Integral area	17885	510148	38312	36725	
LNU-45	Relative content	2.97%	84.59%	6.35%	6.09%	13.32
CTL adsorbed	Integral area	18354	367212	39254	45753	
LNU-45	Relative content	3.90%	78.04%	8.34%	9.72%	9.35
DCN adsorbed	Integral area	20457	479217	38325	42743	
LNU-45	Relative content	3.52%	82.52%	6.60%	7.36%	12.50
TFL adsorbed	Integral area	1985	498318	29187	80153	
LNU-45	Relative content	0.33%	81.73%	4.79%	13.15%	17.06

Table S3. Element analysis based on the integral area of XPS spectra (Figure S7c).

	Element	Cl_{2p}	C_{1s}	N_{1s}	F_{1s}	C/N
	Binding energy (eV)	189 – 202	280 – 295	395 – 410	680 – 695	ratio
LNU-45	Integral area	1916	413817	24267	40231	
	Relative content	0.40%	86.17%	5.05%	8.38%	17.06
NF adsorbed	Integral area	18442	508278	39145	36923	
LNU-45	Relative content	3.06%	84.32%	6.49%	6.13%	12.98
CTL adsorbed	Integral area	18924	371788	39476	46314	
LNU-45	Relative content	3.97%	78.03%	8.28%	9.72%	9.42
DCN adsorbed	Integral area	21078	472358	38973	43071	
LNU-45	Relative content	3.79%	84.88%	7.00%	7.73%	12.12
TFL adsorbed	Integral area	2037	496728	29558	80734	
LNU-45	Relative content	0.33%	81.56%	4.85%	13.26%	16.81

Table S4. Element analysis based on the integral area of XPS spectra (Figure S7d).

	Element	Cl_{2p}	C_{1s}	N_{1s}	F_{1s}	C/N
	Binding energy (eV)	189 – 202	280 – 295	395 – 410	680 – 695	ratio
LNU-45	Integral area	2074	434568	27218	42704	
	Relative content	0.41%	85.79%	5.37%	8.43%	15.97
NF adsorbed	Integral area	20425	525871	39782	38312	
LNU-45	Relative content	3.27%	84.23%	6.37%	6.13%	13.22
CTL adsorbed	Integral area	19861	374589	39541	47536	
LNU-45	Relative content	4.12%	77.80%	8.21%	9.87%	9.47
DCN adsorbed	Integral area	24515	416863	39754	45872	
LNU-45	Relative content	4.66%	79.10%	7.54%	8.70%	10.49
TFL adsorbed	Integral area	1572	508151	30742	80468	
LNU-45	Relative content	0.27%	81.83%	4.95%	12.95%	16.53

Table S5. CHN element analysis.

	C	N	H	C/N ratio
LNU-45	84.07%	5.18%	1.27%	16.23
NF adsorbed LNU-45	82.39%	6.27%	1.58%	13.14
CTL adsorbed LNU-45	78.55%	8.35%	2.85%	9.41
DCN adsorbed LNU-45	87.16%	7.65%	1.78%	11.39
TFL adsorbed LNU-45	88.67%	5.36%	1.54%	16.54

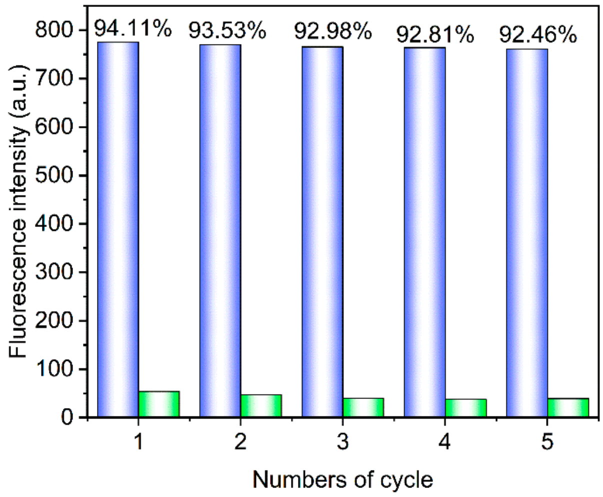

Figure S8. Five consecutive quench/regeneration cycles of LNU-45. The blue and green columns represent the fluorescent intensity of original status and treated with DCN, respectively.

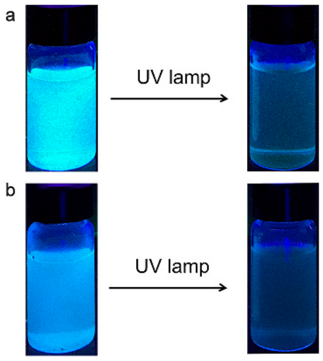

Figure S9. Photographs showing the quenching process of DCN for LNU-45 (**a**) and LNU-47 (**b**).

Reference:

[1] Shard, A.G.; Spencer, S.J. *Surf. Interface Anal.* **2017**, *49*, 1256–1270.

[2] Müller, A.; Sparnacci, K.; Unger, W.E.S.; Tougaard, S. Determining nonuniformities of core-shell nanoparticle coatings by analysis of the inelastic background of X-ray photoelectron spectroscopy survey spectra. *Surf. Interface Anal.* **2020**, *52*, 770–777.

[3] Oku, M.; Shishido, T.; Matsuta, H.; Wagatsuma, K. Comparison of the background corrected valence band XPS spectra of Fe and Co aluminides and silicides with their electronic structures. *J. Electron. Spectros. Relat. Phenomena* **2006**, *153*, 75–80.

[4] Turner, N.H. Estimates of peak areas and relative atomic amounts from wide-scan XPS spectra. *Surf. interface anal.* **1992**, *18*, 47–51.

[5] Dwyer, V.M. Background intensity determination in AES/XPS. *Surf. Sci.* **1988**, *193*, 549–568.

[6] Engelhard, M.H.; Baer, D.R.; Herrera-Gomez, A.; Sherwood, P.M.A. Introductory guide to backgrounds in XPS spectra and their impact on determining peak intensities. *J. Vac. Sci. Technol. A* **2020**, *38*, 063203–063227.

[7] Tougaard, S. Practical guide to the use of backgrounds in quantitative XPS. *J. Vac. Sci. Technol. A* **2021**, *39*, 011201–011222.
